# NDB-UFES: An oral cancer and leukoplakia dataset composed of histopathological images and patient data

**DOI:** 10.1016/j.dib.2023.109128

**Published:** 2023-04-07

**Authors:** Maria Clara Falcão Ribeiro-de-Assis, Júlia Pessini Soares, Leandro Muniz de Lima, Liliana Aparecida Pimenta de Barros, Tânia Regina Grão-Velloso, Renato A. Krohling, Danielle Resende Camisasca

**Affiliations:** a School of Dentistry, Clinical Dentistry Departament, Federal University of Espirito Santo, Vitoria, Brazil; b Nature-inspired Computing Lab, Federal University of Espirito Santo, Vitoria, Brazil; c Graduate Program in Computer Science, Federal University of Espirito Santo, Vitoria, Brazil; d Graduate Program in Science Dentistry, Federal University of Espirito Santo, Vitoria, Brazil

**Keywords:** Mouth diseases, Squamous Cell carcinoma, Oral leukoplakia, Dataset, Information sources

## Abstract

The gold standard for the diagnosis of oral cancer is the microscopic analysis of specimens removed preferentially through incisional biopsies of oral mucosa with a clinically detected suspicious lesion. This dataset contains captured histopathological images of oral squamous cell carcinoma and leukoplakia. A total of 237 images were captured, 89 leukoplakia with dysplasia images, 57 leukoplakia without dysplasia images and 91 carcinoma images. The images were captured with an optical light microscope, using 10x and 40x objectives, attached to a microscope camera and visualized through a software. The images were saved in PNG format at 2048 × 1536 size pixels and they refer to hematoxylin-eosin stained histopathologic slides from biopsies performed between 2010 and 2021 in patients managed at the Oral Diagnosis project (NDB) of the Federal University of Espírito Santo (UFES). Oral leukoplakias were represented by samples with and without epithelial dysplasia. Since the diagnosis considers socio-demographic data (gender, age and skin color) as well as clinical data (tobacco use, alcohol consumption, sun exposure, fundamental lesion, type of biopsy, lesion color, lesion surface and lesion diagnosis), this information was also collected. So, our aim by releasing this dataset NDB-UFES is to provide a new dataset to be used by researchers in Artificial Intelligence (machine and deep learning) to develop tools to assist clinicians and pathologists in the automated diagnosis of oral potentially malignant disorders and oral squamous cell carcinoma.

**Specifications Table**

**Table:** 

Subject	Oral Pathology, Cancer Research, Computer vision, Artificial Intelligence

Specific subject area	Automated distinction from oral squamous cell carcinoma and epithelial dysplasia (oral leukoplakia) using deep learning on histopathological slide images.
Type of data	Images and metadata
How the data were acquired	Firstly, lesions (oral leukoplakia and squamous cell carcinoma) were submitted to oral biopsies and usual histological processing. Histopathological slides were obtained and stained with hematoxilin and eosin. Final diagnosis was reached by consensus among two or three oral pathologists, associating all data available. Images were taken from representative areas of the lesions, using a camera attached to the Leica DM500 Microscope (Heerbrugg, Switzerland) and Leica ICC50 HD Microscope Camera (Heerbrugg, Switzerland), with 10x and 40x objectives. Demographic and clinical data were extracted from histopathological requisitions and/or patient's files and charts, stored in Excel.
Data format	Raw Analyzed
Description of data collection	Each sample in this dataset consists of at least two histopathological images, sociodemographic (year of first care, biopsy date, patient age and skin color), and clinical data (tobacco use, alcohol consumption, sun exposure, fundamental lesion, type of biopsy, lesion color, lesion surface and lesion diagnosis). Inclusion criteria were: all patients seen at the Oral Diagnosis project (NDB-UFES) from 2010 to 2021 who were diagnosed with oral leukoplakia or squamous cell carcinoma after histopathological analysis. Exclusion criteria were: patients with incomplete diagnostic data and patients whose histopathological slides could not be retrieved or could not be analyzed.
Data source location	Institution: Federal University of Espírito Santo (UFES) Vitória – Espírito Santo. Brazil
Data accessibility	Repository name: NDB-UFES: An oral cancer and leukoplakia dataset composed of histopathological images and patient data Dataset is available on [http://doi.org/10.17632/bbmmm4wgr8] Direct URL to data:[https://data.mendeley.com/datasets/bbmmm4wgr8]
Related research article	de Lima LM, de Assis MCFR, Soares JP, Grão-Velloso TR, de Barros LAP, Camisasca DR, Krohling RA. On the importance of complementary data to histopathological image analysis of oral leukoplakia and carcinoma using deep neural networks. Intelligent Medicine, 2023. Available at: [https://doi.org/10.1016/j.imed.2023.01.004]

## Value of the Data

• Automated detection of epithelial dysplasia and squamous carcinoma of the oral cavity through histopathological images can help reduce intra and interobserver diagnostic disagreement, especially with regard to borderline situations, such as the absence of epithelial dysplasia and the presence of mild dysplasia; or severe dysplasia versus microinvasive carcinoma, assisting the pathologist and making the diagnostic process more accurate.
• This dataset is an effort to help researchers develop tools, in particular, to aid in the detection of oral cavity cancer, and ultimately, to increase awareness of possible morphological factors involved in malignant transformation.
• This dataset may be used to support research in deep and machine learning aiming to develop automated tools (CAD) to detect the presence of oral epithelial dysplasia as well as oral cavity cancer (oral squamous cell carcinoma) using histopathological slide imaging in data training and validation.
• In addition to the histopathological images captured, this dataset also contains the patient's socio-demographic and clinical data related to each image, which can help researchers understand the relationship between these data and how they can be used in association, to improve the detection of oral squamous cell carcinoma or aiding in the prediction of malignant transformation.
• The data may be useful for educational purposes, i.e. to train dental students or to standardize specialists in oral pathology from the same center regarding the diagnosis of oral epithelial dysplasia and oral squamous cell carcinoma.

## 1 Objective

A dataset of both oral squamous cell carcinoma (OSCC) and oral leukoplakia that can help artificial intelligence/machine learning algorithms to classify/differentiate a malignant lesion from its most common potentially malignant counterpart.

A start-point dataset to help comprehend histopathological changes that may lead to malignant transformation.

This data was applied leading to an original article publication [1] which showed that sociodemographic and clinical information positively influence the performance of artificial intelligence models when using histopathological image analysis and deep neural network.

## 2 Data Description

**Fig. 1:**
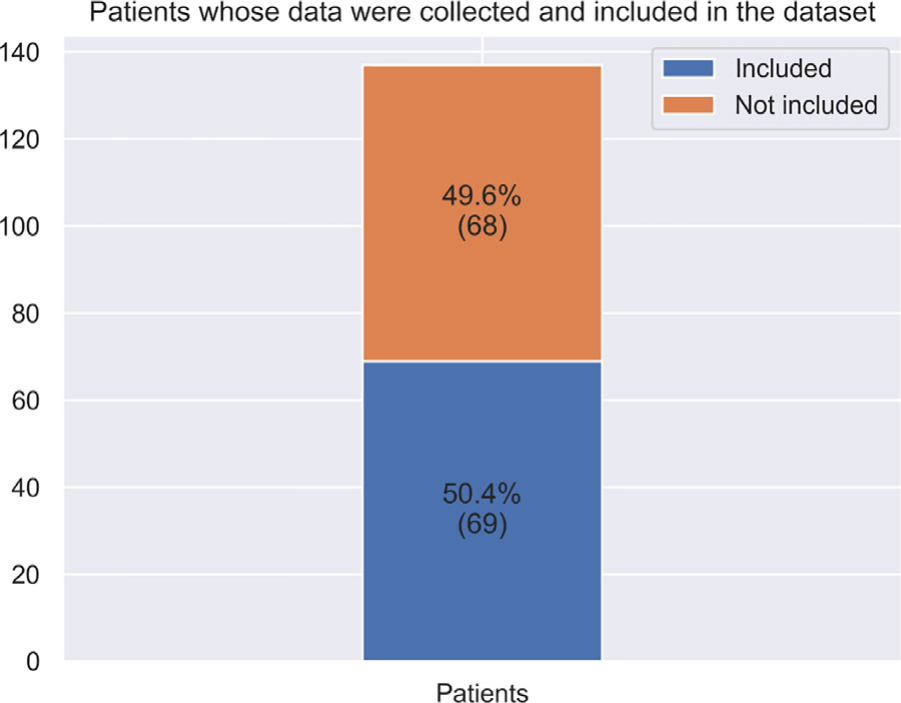
Descriptive analysis of the data, based on percentages of patients initially included and registered in the present dataset.

**Fig. 2:**
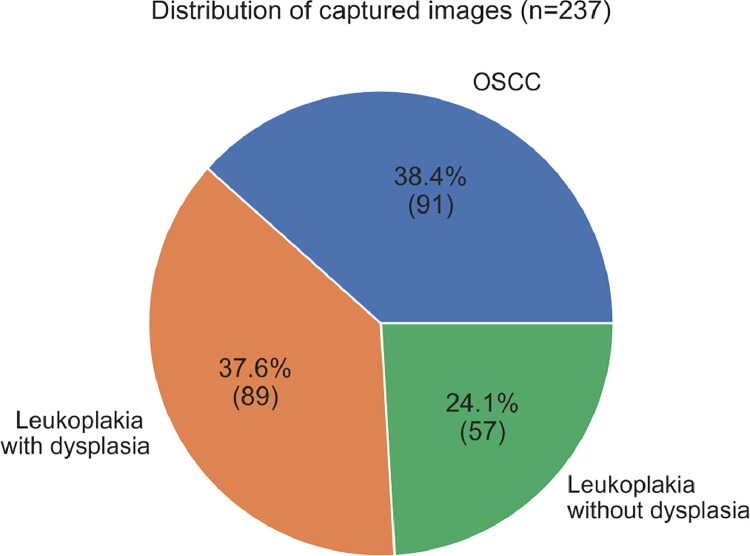
Descriptive analysis of the data, according to the distribution of captured images (*n* = 237).

All cases within this dataset are represented by a patient with at least one lesion in the oral mucosa, from which two or more histopathological images were captured, in addition to a set of metadata associated.. A patient may show one or more mouth lesions and a mouth lesion may have two or more images. In total, 137 patients were identified and data were collected from 69 patients so far (Fig. 1). From the recovered slides, a total of 237 images were captured with the 10x and 40x objectives of a light microscope Leica DM500 (Heerbrugg, Switzerland) and Leica ICC50 HD Microscope Camera (Heerbrugg, Switzerland), LAS EZ software (Leica Application Suite 2.0.0, Heerbrugg, Switzerland), 91 from oral squamous cell carcinoma and 146 from oral leukoplakia (89 with epithelial dysplasia and 57 without epithelial dysplasia) (Fig. 2). For the context of oral lesions, there are currently few public datasets available [2]. Although the total sample is not as high as that of Rahman et al. [3], [4], with 1224 images, demographic and clinical data are associated with the images, which were not previously included in any other previous publicly available database involving malignant neoplasms and oral potentially malignant disorders, to the best of our knowledge our dataset is the first archive containing histopathological images and related demographic and clinical data (XLS format). Further, this dataset contains oral squamous cell carcinoma images, and also oral leukoplakia images, along with the information on the presence or absence or epithelial dysplasia (Data is presented as Portable Network Graphics (PNG) saved in .jpg format at 2048 × 1536 pixels).

The last archive consists of patch images, obtained from the original ones, that are 512 × 512 pixels in PNG format. In a total of 3763 patches, out of these, 1930 (51.29%) were classified as with dysplasia, 1126 (29.92%) as carcinoma, and 707 (18.79%) as without dysplasia.

**Fig. 3:**
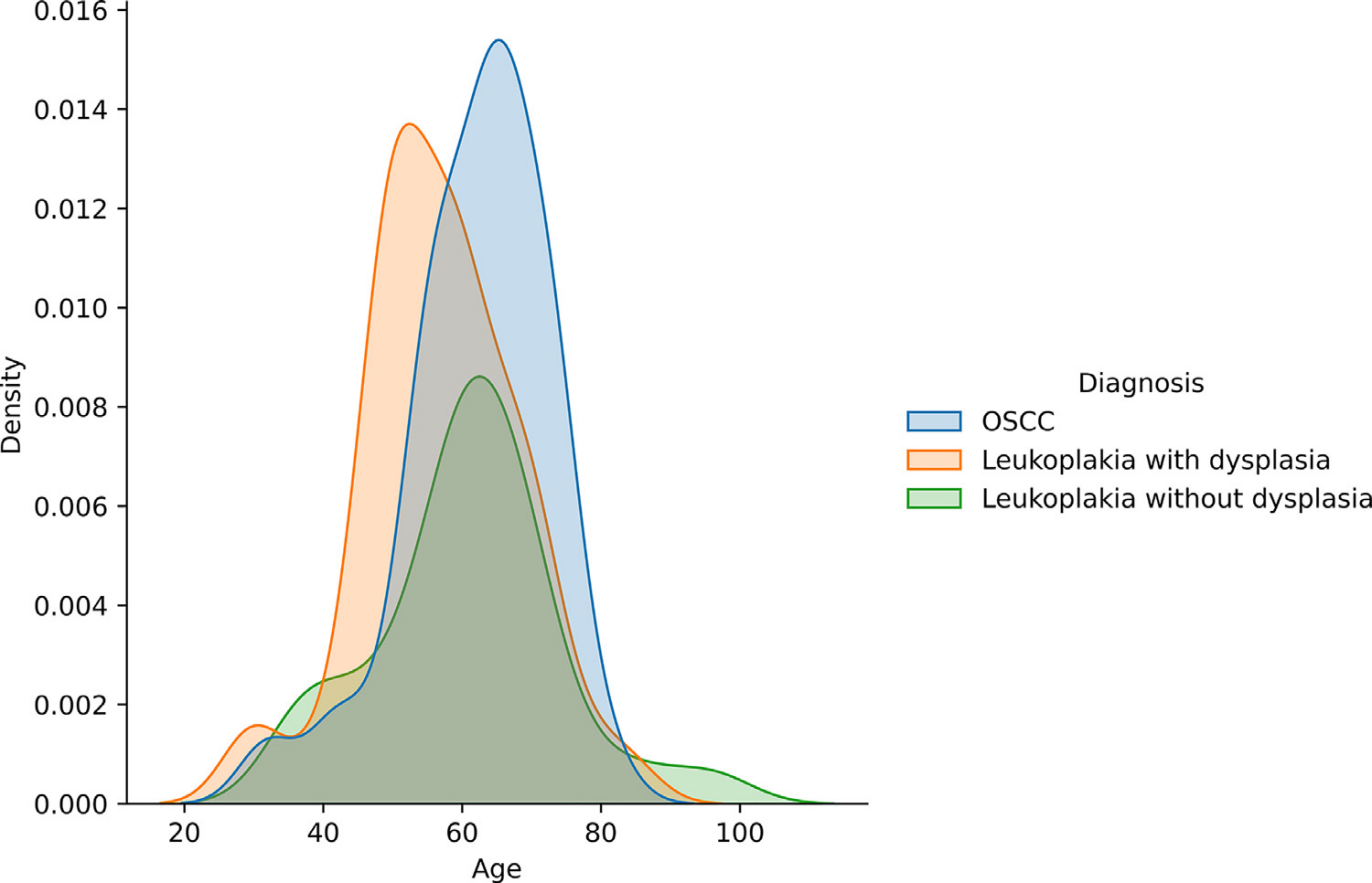
Descriptive analysis of the data, showing age distribution according to lesion type.

The amount of lesions included were 47 from leukoplakias and 30 from OSCC, totalizing 77 lesions. Each patient could have one or more lesions, and each patient could have been submitted to one or more biopsies with their respective histopathological slides. Among the 69 patients recovered, 56,53% were men, and those who reported their skin color, 34,78% were white people. Most of them (53,63%) were more than 60 years old, and three age groups (<40, 41-60 and >61 years old) were stablished, because 40 year-old patients or younger are considered young and patients older than 60 years are considered elders, when there is an elevated chance to develop cancer, due to prolonged exposition to risk factors. Age was also analyzed according to lesion type (Fig. 3). Considering risk factors, 27,53% were tobacco users and 5,8% were former tobacco users. Few of them used alcoholic beverages (13,05%) or used to drink alcohol beverages in the past (11,6%) (Table 1).

**Table 1:** Distribution of lesions by definitive diagnosis and patients by gender, age, skin color, and risk factors (alcohol and tobacco use).

Variable	[n]	[%]	
Diagnosis	Oral squamous cell carcinoma	30	38,96
	Leukoplakia	47	61,04
	**Total**	**77**	100
Gender	Female	30	43,47
	Male	39	56,53
	**Total**	**69**	**100**
Age group	< 40	2	2,9
	41 – 60	30	43,47
	> 60	37	53,63
	**Total**	**69**	**100**
Skin color	Brown	6	8,7
	Black	9	13,05
	White	24	34,78
	Not informed	30	43,47
	**Total**	**69**	**100**
Tobacco use	Yes	19	27,53
	Former	4	5,8
	No	11	15,95
	Not informed	35	50,72
	**Total**	**69**	**100**
Alcohol consumption	Yes	9	13,05
	Former	8	11,6
	No	17	24,63
	Not informed	35	50,72
	**Total**	**69**	**100**

## 3 Experimental Design, Materials and Methods

### 3.1 Data Collection

**Fig. 4:**
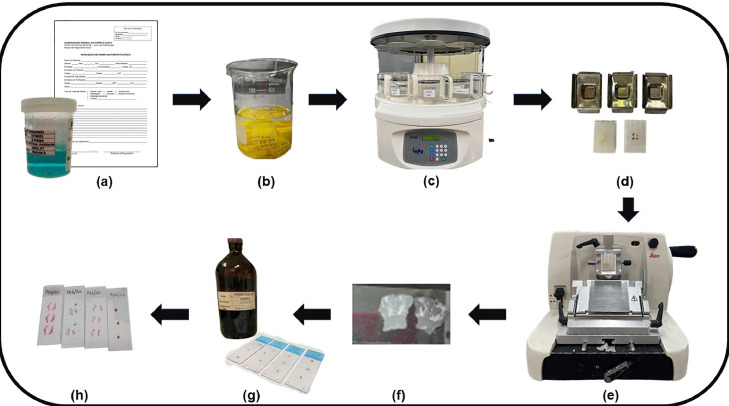
Histological processing of tissues obtained by biopsy. [a] Conditioning of the sample removed by biopsy in a vial containing formalin and sent with the biopsy requisition form filled in with the patient and lesion data. [b] Fixation of the sample in 10% formalin for at least 6 hours. [c] Dehydration in alcoholic solutions at increasing concentrations, followed by clarification with xylene. [d] Embedding the sample in paraffin. [e] Cutting the paraffin blocks with a microtome. [f] Insertion of the cut into the slide. [g] Deparaffinization and staining with hematoxylin and eosin. [h] Slides are then ready for observation using a microscope.

Patients are examined at the NDB-UFES to diagnose and treat numerous mouth diseases. Students under the supervision of oral medicine and oral pathology professors assist the patients and evaluate the oral lesion through clinical examination. If there is a need for biopsy, the patient is referred to the Oral and Maxillofacial Surgery Service, the tissue sample is then sent to the Oral Pathological Anatomy Service SAP-UFES for histopathological examination. After surgical removal, the specimen is processed to obtain histopathological slides for microscopic analysis (Fig. 4). On the other hand, it is common that certain mouth diseases do not need histopathological analysis for definitive diagnosis. This is decided during the diagnostic process, analyzing clinical exam information along with the history of current illness, as well as imaging exams and/or serological tests. All samples received at SAP-UFES are accompanied by histopathological requisitions with clinical and sociodemographic data of the patient, in addition to a brief summary of the history of current illness and lesion clinical description.

### 3.2 Data Selection

Data were collected from all patients diagnosed with oral leukoplakia and oral squamous cell carcinoma from January 2010 to December 2021 in the extension project Service of Oral Pathological Anatomy of the Dentistry Course at UFES. Cases in which it was not possible to retrieve the histopathological slides or paraffin embedded blocks, as well as cases in which there was not enough material for histopathological analysis, were excluded.

The NDB-UFES extension project routinely assists patients with oral diseases and tissues obtained through biopsies or surgical removal with curative intent are sent for microscopic analyzes at the SAP-UFES. Clinical and demographic data, as well as histopathological reports are routinely typed into the Oral Analysis Software, which is used for data record and organization. Cases that were not yet registered in the software were inserted so that there was the possibility of data analysis.

The NDB dataset consist of histopathological images and the demographic data (year of biopsy, date of biopsy, gender, age of the patient, skin color) as well as clinical data (tobacco use, alcohol consumption, type of lesion, site of the lesion, type of biopsy, lesion color, lesion surface and lesion diagnosis) and histopathological images.

### 3.3 Histological Processing and Image Capture

**Fig. 5:**
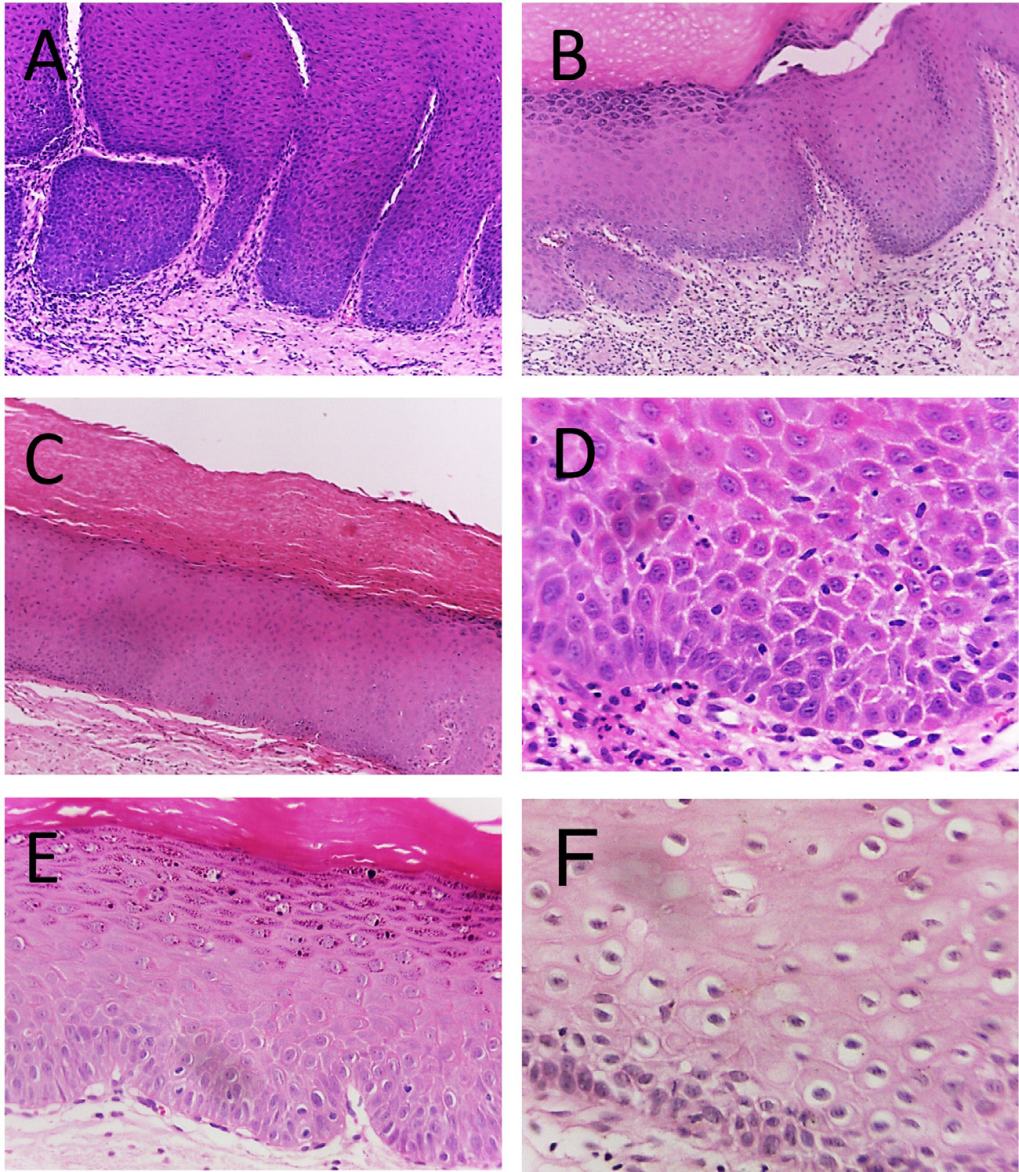
[A - C] Oral leukoplakias histopathological images obtained with the 10x objective lens of a light microscope. [A] Hyperplastic squamous epithelium is observed, showing intense acanthosis, in addition there is a subepithelial inflammatory infiltrate. [B] Evident hyperorthokeratosis, with acquisition of the granular layer and acanthosis, there are vessels and inflammatory infiltrate in the connective tissue. [C] Thick orthokeratinized layer present on the surface of the epithelium; also a flat interface with connective tissue is seen. [D - F] Histopathological images of oral leukoplakias, captured with the 40x objective lens. [D] Intercellular bridges in the epithelium, exocytosis, and mild cellular pleomorphism are observed. In the connective tissue there is lymphocytic inflammatory infiltrate. [E] Hyperplastic orthokeratinized epithelium, with evident granular layer, and epithelial dysplasia in the lower third, evidenced by the presence of drop-shaped rete ridges along with nuclear and cellular pleomorphism. [F] Presence of koilocytosis.

After surgical removal of the lesion or a part of it, as an incisional or excisional biopsy, the tissue is placed in a vial with 10% formalin or buffered formalin, identified and sent for histopathological analysis, along with the requisition form (Fig. 5a). After fixation for at least 6 hours (Fig. 5b), the tissues are processed at the Multiuser Laboratory of Histotechnics (Laboratório de Histotécnicas Multiusuários - CCS/UFES), through dehydration in a series of alcoholic solutions at different concentrations (Fig. 5c), followed by clarification using xylene, to finally be embedded in paraffin (Fig. 5d). The paraffin blocks were then cut on microtomes (Fig. 5e) and the 5-micrometer sections placed on a glass slide (Fig. 5f), deparaffinized and stained with hematoxylin and eosin (Fig. 5g). The sections are covered with a coverslip and observed under a light microscope (Fig. 5h).

**Fig. 6:**
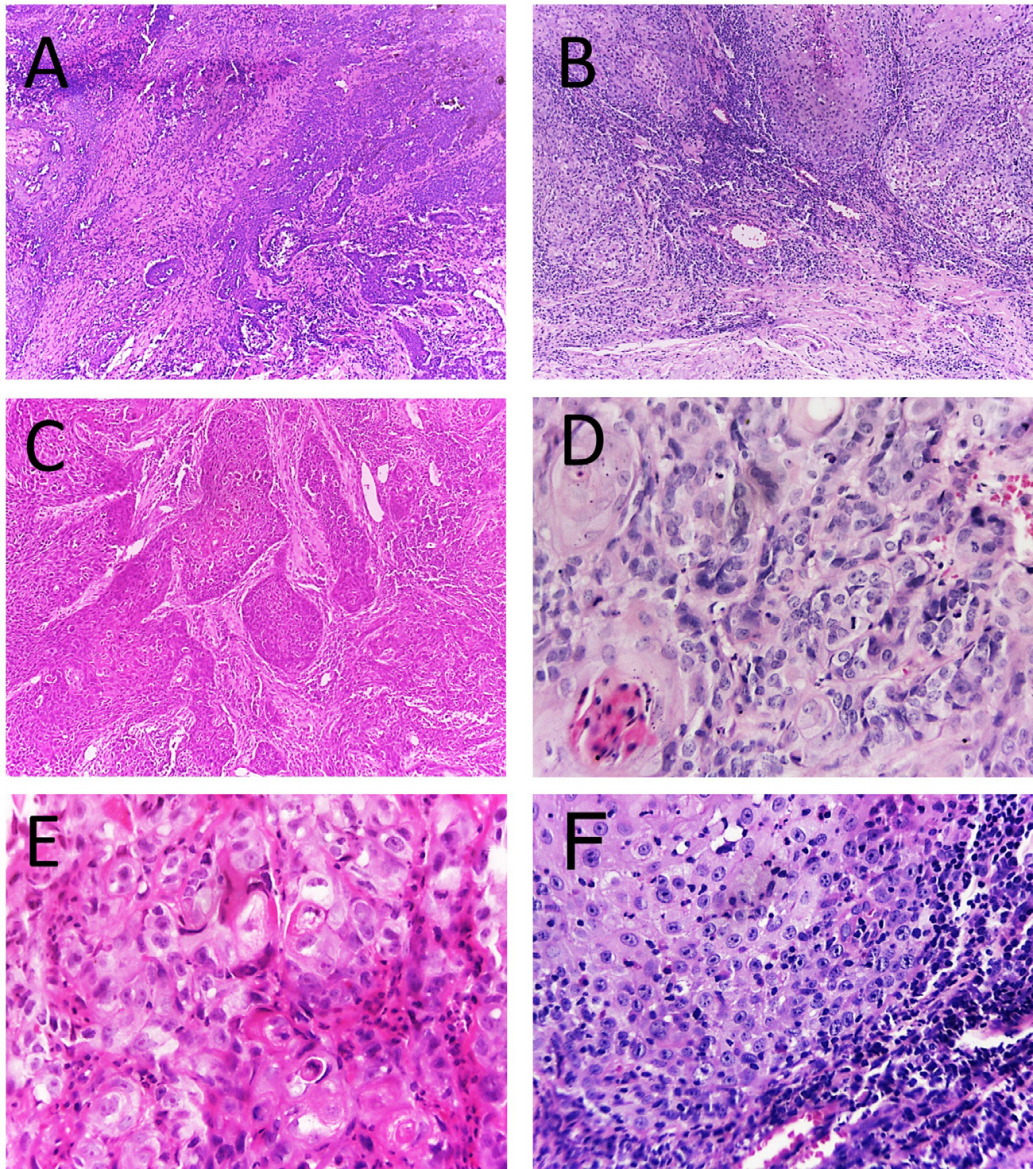
[A - C] Oral squamous cell carcinoma histopathological images obtained with a 10x objective lens of a light microscope. [A] Cords of epithelial cells invading connective tissue with intense surrounding inflammatory infiltrate.[B] Islands of epithelium beginning to invade connective tissue which shows a band of inflammatory infiltrate. [C] Islands and cords of neoplastic epithelium in frank invasion of connective tissue. [D - F] Oral squamous cell carcinoma histopathological images captured with a 40x objective lens of a light microscope. [D] Presence of pleomorphic cells with evident nucleoli, presence of a keratin pearl (white arrow). [E] Presence of intense cellular pleomorphism. [F] Epithelial island with exocytosis circumscribed by intense lymphocytic inflammatory infiltrate.

From the previously included cases, representative slides were selected to capture histopathological images using a camera attached to the Leica DM500 Microscope (Heerbrugg, Switzerland), Leica ICC50 HD Microscope Camera (Heerbrugg, Switzerland) and software LAS EZ (Leica Application Suite 2.0.0, Heerbrugg, Switzerland), with 10x and 40x objectives (Figs. 5 and 6). The images were saved as Portable Network Graphics (PNG), at a size of 2048 × 1536 pixels. Images were captured from the worst area of the lesion (considering worst dysplasia degree or worst tumor grading) or from the most prevalent area, according to the features present in each histopathological slide.

The images included in this paper belong to cases of oral leukoplakia and oral squamous cell carcinoma obtained from patients attended at UFES. Microscopic analysis is routinely performed by two or three oral pathologists that reach histopathological diagnosis in consensus, taking in consideration sociodemographic, clinical and image data in association with histopathological data. After inclusion in the study, a training session was performed to teach the junior researcher how to select the appropriate area to be registered from the histologic slides. This session was under supervision of an oral pathologist with expertise in oral cancer. In this sense, the junior researcher selected representative areas of the lesions. As stated above, the main diagnosis was previously set by two or three pathologists in consensus and upon image registration, the researcher was aware of the diagnosis, in order to register areas representative of the whole lesion. After histologic images capture and registration, one oral pathologist reviewed the images to verify their quality.

Another steps of the study consisted of the analysis of smaller parts, known as patches, of the captured images from oral squamous carcinomas, and oral leukoplakia. In this new scenario, images of slides retrieved between the years 2011 and 2021 were used and segmented. After segmentation, each patch was once again classified, according to the histopathological features shown in that specific region. Then, the patch was labeled as with or without dysplasia, or as carcinoma. The patch images are 512 × 512 pixels in PNG format. In a total of 3763 patches, out of these, 1930 (51.29%) images were classified as with dysplasia, 1126 (29.92%) as carcinoma, and 707 (18.79%) as without dysplasia. For this classification, images were evaluated again by two observers with the previous knowledge of the diagnosis (leukoplakia or carcinoma), once a very small part of the lesion sometimes does not allow for correct interpretation of the features necessary to consider it a carcinoma, such as invasion of the connective tissue. Also, blank or blurry images were excluded.

## Ethics Statements

The present study was approved by the Research Ethics Committee of the Hospital Universitário Cassiano Antonio de Moraes da Universidade Federal do Espírito Santo under registration no. 5,022,438.

## CRediT authorship contribution statement

**Maria Clara Falcão Ribeiro-de-Assis:** Data curation, Formal analysis, Writing – original draft, Writing – review & editing. **Júlia Pessini Soares:** Data curation, Writing – review & editing. **Leandro Muniz de Lima:** Software, Writing – review & editing. **Liliana Aparecida Pimenta de Barros:** Data curation, Formal analysis, Writing – review & editing. **Tânia Regina Grão-Velloso:** Data curation, Formal analysis, Writing – review & editing. **Renato A. Krohling:** Conceptualization, Funding acquisition, Writing – review & editing. **Danielle Resende Camisasca:** Conceptualization, Methodology, Data curation, Funding acquisition, Project administration, Formal analysis, Writing – review & editing, Supervision.

## Declaration of Competing Interest

The authors declare that they have no known competing financial interests or personal relationships that could have appeared to influence the work reported in this paper.

## Data Availability

NDB-UFES: An oral cancer and leukoplakia dataset composed of histopathological images and patient data (Original data) (Mendeley Data).

## References

[1] de Lima LM, de Assis MCFR, Soares JP, Grão-Velloso TR, de Barros LAP, Camisasca DR, Krohling RA. (2023). On the importance of complementary data to histopathological image analysis of oral leukoplakia and carcinoma using deep neural networks. Intell. Med..

[2] Sengupta N, Sarode SC, Sarode GS, Ghone U. (2022). Scarcity of publicly available oral cancer image datasets for machine learning research. Oral Oncol..

[3] Rahman TY, Mahanta LB, Das AK, Sarma JD. (2020). Histopathological imaging database for oral cancer analysis. Data Br..

[4] Rahman TY, Mahanta LB, Das AK, Sarma JD. (2020). Automated oral squamous cell carcinoma identification using shape, texture and color features of whole image strips. Tissue Cell.

